# An affordable approach to classifying type 2 diabetes based on fasting plasma glucose, TyG index and BMI: a retrospective cohort study of NHANES Data from 1988 to 2014

**DOI:** 10.1186/s13098-022-00883-0

**Published:** 2022-08-10

**Authors:** Jing Xie, Xin Zhang, Hua Shao, Shenqi Jing, Tao Shan, Yaxiang Shi, Yong Li, Yun Liu, Naifeng Liu

**Affiliations:** 1 College of Basic Medicine and Clinical Pharmacy, China Pharmaceutical University, Nanjing, 210009 Jiangsu China; 2 Department of Information, The First Affiliated Hospital, Nanjing Medical University, Nanjing, 210029 Jiangsu China; 3 Department of Medical Informatics, School of Biomedical Engineering and Informatics, Nanjing Medical University, Nanjing, 211166 Jiangsu China; 4 Department of Pharmacy, School of Medicine, Zhongda Hospital, Southeast University, Nanjing, 210009 Jiangsu China; 5 Department of Outpatient, The First Affiliated Hospital, Nanjing Medical University, Nanjing, 210029 Jiangsu China; 6 Department of Information, School of Medicine, Zhongda Hospital, Southeast University, Nanjing, 210009 Jiangsu China; 7 Department of Cardiology, The First Affiliated Hospital, Nanjing Medical University, Nanjing, 210029 Jiangsu China; 8 Institute of Medical Informatics and Management, Nanjing Medical University, Nanjing, 210029 Jiangsu China; 9 Department of Cardiology, School of Medicine, Zhongda Hospital, Southeast University, Nanjing, 210009 Jiangsu China

**Keywords:** Type 2 diabetes, Affordable, Predictability, Subgroup

## Abstract

**Background:**

The β-cell function and insulin resistance required by existing methods of classifying type 2 diabetes are not routinely adopted in most medical institutions of developing countries and regions. This study aims to propose a novel, affordable classification approach and evaluate its predictive ability for several health and mortality outcomes, including cardiovascular health (CVH), retinopathy, chronic kidney disease (CKD), nonalcoholic fatty liver disease (NAFLD), advanced liver fibrosis, and mortality caused by all-cause, cardiovascular disease (CVD), cancer.

**Methods:**

Based on 4060 participants with diabetes (aged ≥ 30 at the time of diagnosis) selected from the National Health and Nutrition Examination Survey III & 1999–2014, we proposed a novel, but simple classification approach based on the threshold of fasting plasma glucose (FPG), triglyceride-glucose (TyG) index and body mass index (BMI). We used logistic regression model to assess its predictability for diabetes complications, and Cox regression model to estimate the mortality risks.

**Results:**

By utilizing this approach, we characterized the subjects into four subgroups: subgroup A (obesity-related), which accounts for 37% of the total, subgroup B (age-related), 38%, subgroup C (insulin resistance), 20%, and subgroup D (severe insulin deficiency), 5%. Subjects in subgroup D had a higher risk of retinopathy, in subgroup B had a lower risk of poor cardiovascular health, nonalcoholic fatty liver disease, and advanced liver fibrosis, in subgroup C had a higher risk of all-cause mortality.

**Conclusions:**

This study proposes an affordable and practical method for classifying patients with type 2 diabetes into different subgroups, with a view to yield a high predictability of patient outcomes and to assist clinicians in providing better treatment.

**Supplementary Information:**

The online version contains supplementary material available at 10.1186/s13098-022-00883-0.

## Introduction

Diabetes is increasingly prevalent globally, particularly in developing countries and regions. According to the International Diabetes Federation (IDF), 79% of diabetes patients live in low and middle-income countries (https://www.diabetesatlas.org/en/resources/). Type 2 diabetes (T2D), as the primary type of diabetes, is highly heterogeneous in clinical characteristics, progression and risk of complications [1]. Through targeted prevention and treatment, we could prevent diabetes complications and reduce the risk of premature death. Given its heterogeneity, attempts have been made to define its subgroups through genetic [2, 3] or clinical features [4–9]. Although genetic data are stable over a patient’s lifetime and not influenced by disease progression, they are often not available. Based on clinical features, the majority of current studies used five clinical variables (age, BMI, hemoglobin A1c, homeostasis model assessment of β cell function, and homeostasis model assessment of insulin resistance) to stratify T2D patients into severe insulin-deficient diabetes (SIDD), severe insulin-resistant diabetes (SIRD), mild obesity-related diabetes (MOD), and mild age-related diabetes (MARD) [5, 10]. Instead of k-means clustering method, Li and Chen used thresholds for HOMA2-IR, HOMA2-β and BMI to separate the T2D subgroups, which is a more convenient method in clinical applications [11].

However, islet function and insulin resistance required for these classification methods are expensive, hard to be standardized, and not routinely adopted in most medical institutions of developing countries and regions, which means such classification methods are unfeasible for two-thirds of the world’s population with diabetes [12]. Therefore, alternative variables which are affordable and readily available are needed to detect their insulin resistance and islet function. The triglyceride-glucose (TyG) index as an alternative variable under fasting state is affordable and easily available. It has been described as a biochemical marker of insulin resistance [13–15]. In addition, according to previous studies, there was a significant relationship between β-cell mass and fasting plasma glucose (FPG) concentration [16, 17]. Gordon C. Weir and Susan Bonner-Weir proposed five stages of diabetes progression by FPG levels, each characterized by different changes in the mass, phenotype and function of β cells. At stage 4 and 5, patients’ islet function begins to decline gravely [18].

Against this background, we proposed to generate subgroups based on established thresholds for low-cost and high availability of diabetes characteristics, and test whether participants in these subgroups have different risks for several outcomes, including cardiovascular health (CVH), chronic kidney disease (CKD), nonalcoholic fatty liver disease (NAFLD), advanced liver fibrosis, retinopathy, and mortality caused by all-cause, cardiovascular disease (CVD), cancer. The results of this study will enable targeted therapeutic interventions for people in developing countries and regions.

## Methods

### Subjects

**Fig. 1:**
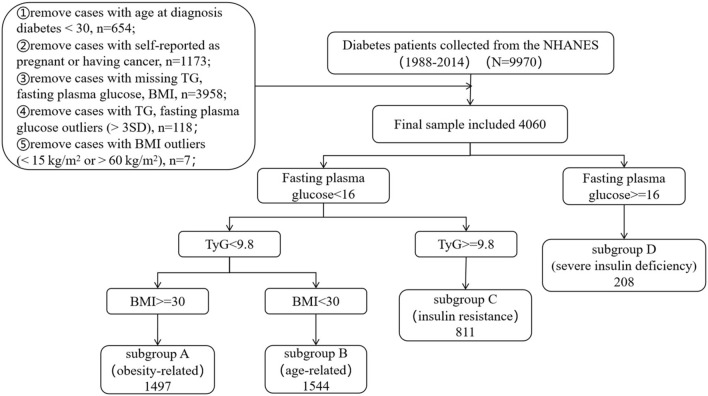
Algorithm for Type 2 diabetes selection in the NHANES (1988–2014)

We used data from 1988 to 1994 and 1999 to 2014 of the National Health and Nutrition Examination Survey (NHANES). Diabetes is defined as a self-reported diagnosis, use of insulin or oral hypoglycemic medication, FPG ≥ 7.0 mmol/L or glycated haemoglobin (HbA1c) level ≥ 6.5%, according to ADAs diabetes diagnostic criteria [19]. To ensure the samples were type 2 diabetes, we excluded patients who were diagnosed with diabetes before the age of 30 years [20]. We also excluded patients who were self-reported as pregnant or having cancer at baseline. Some patients were also excluded according to the following criteria: (1) missing triglycerides (TG), FPG and BMI values at baseline; (2) extreme TG or FPG values (> 3SD) [21]; (3) BMI outliers (< 15 kg/m^2^ or > 60 kg/m^2^) [22]. The details were shown in Fig. 1 and the final sample size was 4,060.

### Definitions of different subgroups of T2D

According to Gordon C. Weir and Susan Bonner-Weir’s five stages of evolving beta-Cell dysfunction in the progression of diabetes, when patients’ fasting plasma glucose levels exceeds 16 mmol/L, their capacity for insulin secretion is considerably less than the case of 50% reduction in β-cell mass, which results in less efficient insulin secretion [18]. Hence, we defined severe insulin-deficient as fasting glucose greater than 16 mmol/L. In previous studies, the 75th percentile of the HOMA-IR level in the population was often used as the threshold of IR [23–25]. As a marker of insulin resistance, the higher the TyG value, the higher the level of insulin resistance. Thus, we defined server insulin resistance as the TyG above the 75th percentile value. The TyG index was calculated by TyG index = Ln [fasting TG (mg/dL) × fasting glucose (mg/dL)/2]. According to the World Health Organization (WHO), obesity is defined as BMI ≥ 30 kg/m^2^. The grouping rules were shown in Fig. 1. Acknowledging differences in the approach of this study and the study by Ahlqvist [5] and Peng-Fei Li et al. [11], we chose letter-based grouping labels, which correspond to the replicated Ahlqvist et al. labels as follows: subgroup A, obesity-related diabetes; subgroup B, age-related diabetes; subgroup C, insulin resistant diabetes, and subgroup D, severe insulin deficient diabetes.

### Outcome assessment

According to the American Heart Association's Life's Simple 7 (LS7) [26], CVH was evaluated based on smoking, weight, physical activity, diet, blood cholesterol, blood glucose, and blood pressure. The definitions of for each metric were shown in Additional file 1: Table S1. Each LS7 component was given a score of 0, 1, or 2 to reflect poor, intermediate, and ideal health, respectively. A total LS7 score between 0 and 14 was calculated as the sum of the LS7 component scores, and poor CVH was defined as a score between 0 and 4 [27]. CKD was defined as kidney damage and an estimated glomerular filtration rate (eGFR) of less than 60 ml min-1 per 1.73 m^2^, and eGFR was calculated using the CKD-EPI study equation with serum creatinine [28]. Fundus Photograph data was only available for 1988–1994, 2005–2008, and the final sample was 2276. Retinopathy was defined as the presence of the following factors on fundus photograph: non proliferative retinopathy, intraretinal microvascular abnormalities without microaneurysms, retinal microaneurysms, hemorrhages, soft exudates, hard exudates, and proliferative retinopathy [29]. The criteria to categorize Nonalcoholic fatty liver disease (NAFLD) included a United States Steatosis Index (USFLI) score of ≥ 30, no excessive alcohol consumption (average ≤ 1 alcoholic drink per day for women & ≤ 2 alcoholic drinks per day for men), negative Hepatitis C antibody, and negative Hepatitis B surface antigen [30]. The formula for USFLI score was shown in Additional file 1: Table S2 [31]. Due to the lack of data on drinking in the 1988–1994, only 1999–2014 was considered, and the final sample was 2343. Advanced liver fibrosis was determined using two noninvasive markers of liver fibrosis: the fibrosis-4 (FIB-4) score and the NAFLD fibrosis score (NFS). Their cutoff values were FIB-4 ≥ 2.67 or NFS > 0.676 [32]. FIB-4 [33] and NFS [34] indexes were calculated as shown in Additional file 1: Table S2.

Mortality data of the NHANES (1988–2014) participants were provided by the National Centre for Health Statistics using probabilistic record matching with death certificate data found in the National Death Index (NCHS Linked Mortality File) by December 31, 2015. Mortality outcomes of interest include all-cause, CVD and cancer-related death. Follow-up period was calculated as the time between the date of NHANES examination date and the last known about each participant’s living or death [35].

### Covariates assessment

Information on age, sex, race/ethnicity, education level, family income, smoking status, physical activity, disease status, and medication use were collected from NHANES household interviews. Measurements of body mass index (BMI), systolic blood pressure (SBP) and diastolic blood pressure (DBP) were obtained in the NHANES MEC. Clinical indicators including fasting glucose, HbA1c, triglycerides (TG), total cholesterol (TC), low-density lipoprotein cholesterol (LDL-C) and high-density lipoprotein cholesterol (HDL-C) were measured in the NHANES laboratory. According to the 2019 ESC/EAS Guidelines, hypertriglyceridemia was defined as triglycerides ≥ 1.7 mmol/L (150 mg/dL) [36]. Education level was categorized as < 9th grade, 9-11th grade, 12th grade and > 12th grade. Family income-to-poverty ratio was classified as 0–1.0, 1.0–3.0, or > 3.0. Smoking status was classified as never smoker, former smoker, or current smoker. Ideal physical activity was defined as ≥ 150 min of moderate-intensity activities per week, ≥ 75 min of vigorous-intensity activities per week, or an equivalent combination of both [37].

### Statistical analysis

Analyses were conducted according to the guidelines recommended by the NHANES, we computed new sample weights according to NCHS guidelines for combining data from multiple cycles. To calculate the differences between different subgroups at baseline, weighted chi-square and linear regression model were used among categorical variables and continuous variables, respectively. Multiple imputation was used for the covariates with missing values. We used odds ratios (ORs) to evaluate the prevalence of poor CVH, CKD, retinopathy, NAFLD and advanced liver fibrosis across different subgroups, and conducted logistic regression to calculate the value of ORs. Individual survival among different subgroups was plotted using Kaplan–Meier curves, and multivariate Cox proportional hazard models were used to obtain the hazard ratios (HRs) of all-cause, CVD and cancer-related mortality. In model 1, we adjusted for age, gender and race/ethnicity. In model 2, we further adjusted for education level, family income-poverty ratio, smoking status, ideal physical activity. In model 3, we further adjusted for duration of diabetes, diabetes medication use, self-reported hypertension, hypercholesterolemia, and CVD, hypertriglyceridemia, self-reported hypertension, hypercholesterolemia medication use, systolic blood pressure, diastolic blood pressure, total cholesterol, high density lipoprotein cholesterol, and low density lipoprotein cholesterol.

Stratified analysis were also conducted by gender (male or female), race/ethnicity (White or non-White), stages of diabetes (newly diagnosed or already diagnosed). The P values for the product terms between subgroups and stratification variables were used to estimate the significance of interactions. As a sensitivity analysis, we additionally performed the analyses after excluding participants who died within 2 years of follow-up, to reduce the potential reverse causation bias. All the analyses were performed in R software (4.1.0).

## Results

### Characteristics of the subgroups

The study included 4060 adults from the NHANES database. Table 1 showed the demographic data of the four subgroups: subgroup A (n = 1497, 36.87%), subgroup B (n = 1544, 38.03%), subgroup C (n = 811, 19.98%), subgroup D (n = 208, 5.12%). As shown in the Table 1, Subgroup A presented the highest BMI, more hypertension and hypertension medication use. Subgroup B was the oldest and had the highest level of HDL. Subgroup C had the highest TG, and relatively high TyG and fasting glucose levels. Subgroup D had the highest fasting glucose, TyG and TC levels, and more diabetes pills and insulin use. Furthermore, subgroup A had the highest prevalence of advanced liver fibrosis, subgroup B had the lowest prevalence of poor CVH. Subgroup D had the highest prevalence of retinopathy. Compared with other subgroups, subgroup C had the highest all-cause and CVD-related mortality.

**Table 1:** Baseline Characteristics of subjects among different T2D subgroups

	Total (n = 4060)	Subgroup A (n = 1497)	Subgroup B (n = 1544)	Subgroup C (n = 811)	Subgroup D (n = 208)	P value
Age (mean ± SE), years	58.13 ± 0.40	56.44 ± 0.53	62.16 ± 0.61	56.24 ± 0.93	52.97 ± 2.00	0.774
FPG (mean ± SE), mmol/L	8.37 ± 0.09	7.51 ± 0.09	7.43 ± 0.09	10.47 ± 0.17	17.70 ± 0.21	< 0.001
TG (mean ± SE), mmol/L	1.96 ± 0.04	1.53 ± 0.03	1.47 ± 0.03	3.77 ± 0.09	2.81 ± 0.24	< 0.001
TyG index (mean ± SE)	9.26 ± 0.03	9.00 ± 0.02	8.95 ± 0.02	10.26 ± 0.03	10.44 ± 0.08	< 0.001
BMI (mean ± SE), kg/m^2^	32.68 ± 0.23	37.07 ± 0.27	26.07 ± 0.12	33.36 ± 0.50	32.59 ± 1.60	< 0.001
Gender						
Male	2133 (52.5)	713 (47.6)	903 (58.5)	455 (56.1)	89 (42.8)	< 0.001
Female	1927 (47.5)	784 (52.4)	641 (41.5)	356 (43.9)	119 (57.2)	
Race/ethnicity						
Non-Hispanic white	2429 (59.8)	939 (62.7)	848 (54.9)	521 (64.3)	91 (43.8)	< 0.001
Non-Hispanic black	647 (15.9)	283 (18.9)	245 (15.8)	69 (8.5)	33 (16.0)	
Non-Hispanic black	389 (9.6)	131 (8.7)	118 (7.7)	109 (13.5)	37 (18.0)	
Other	596 (14.7)	144 (9.6)	333 (21.6)	111 (13.7)	46 (22.2)	
Smoking status						
Never smoker	1978 (48.7)	759 (50.7)	697 (45.2)	385 (47.5)	130 (62.4)	0.238
Ever smoker	1392 (34.3)	486 (32.5)	583 (37.8)	281 (34.6)	50 (23.8)	
Current smoker	690 (17.0)	252 (16.8)	264 (17.1)	145 (17.9)	29 (13.8)	
Education levels						
< 9th grade	462 (11.4)	150 (10.0)	205 (13.3)	90 (11.1)	27 (12.9)	0.168
9-11th grade	689 (17.0)	236 (15.8)	237 (15.4)	170 (20.9)	56 (26.7)	
12th grade	1123 (27.7)	435 (29.0)	427 (27.7)	213 (26.3)	34 (16.4)	
> 12th grade	1786 (44.0)	676 (45.2)	675 (43.7)	337 (41.6)	91 (43.9)	
Family income-poverty ratio						
≤ 1.0	701 (17.3)	244 (16.3)	284 (18.4)	141 (17.4)	40 (19.2)	0.264
1.0–3.0	1800 (44.3)	696 (46.5)	645 (41.8)	335 (41.3)	116 (55.8)	
> 3.0	1559 (38.4)	558 (37.2)	615 (39.8)	335 (41.3)	52 (25.0)	
Ideal physical activity						
Yes	1535 (37.8)	532 (35.5)	625 (40.5)	314 (38.7)	80 (38.5)	0.429
No	2525 (62.2)	965 (64.5)	919 (59.5)	497 (61.3)	128 (61.5)	
Duration of diabetes						
≤ 3 years	1363 (33.6)	540 (36.1)	488 (31.6)	263 (32.4)	50 (24.0)	0.269
3–10 years	1457 (35.9)	536 (35.8)	570 (36.9)	266 (32.8)	91 (43.7)	
> 10 years	1241 (30.6)	421 (28.1)	486 (31.5)	282 (34.8)	67 (32.3)	
Hypertension						
Yes	2463 (60.7)	1030 (68.8)	796 (51.6)	470 (57.9)	107 (51.5)	< 0.001
No	1597 (39.3)	467 (31.2)	748 (48.4)	341 (42.1)	101 (48.5)	
Hypercholesterolemia						
Yes	2106 (51.9)	775 (51.8)	801 (51.9)	431 (53.1)	96 (46.3)	0.876
No	1954 (48.1)	722 (48.2)	743 (48.1)	380 (46.9)	112 (53.7)	
CVD						
Yes	901 (22.2)	338 (22.6)	320 (20.8)	202 (24.9)	34 (16.5)	0.400
No	3159 (77.8)	1159 (77.4)	1224 (79.2)	609 (75.1)	174 (83.5)	
Diabetes medication use						
No insulin or pills	1797 (44.3)	646 (43.2)	716 (46.4)	362 (44.6)	78 (37.3)	0.010
Only diabetes pills	1762 (43.4)	673 (45.0)	669 (43.3)	336 (41.4)	70 (33.6)	
Only insulin	240 (5.9)	77 (5.1)	95 (6.1)	56 (6.9)	19 (9.0)	
Pills and insulin	261 (6.4)	101 (6.8)	64 (4.2)	57 (7.1)	42 (20.1)	
Hypertension medication use						
Yes	2094 (51.6)	891 (59.5)	664 (43.0)	399 (49.2)	80 (38.6)	< 0.001
No	1966 (48.4)	606 (40.5)	880 (57.0)	412 (50.8)	128 (61.4)	
Hypercholesterolemia medication use						
Yes	1515 (37.3)	575 (38.4)	569 (36.9)	300 (36.9)	60 (28.8)	0.668
No	2545 (62.7)	922 (61.6)	975 (63.1)	511 (63.1)	148 (71.2)	
SBP (mean ± SE), mmHg	131.02 ± 0.60	129.58 ± 0.84	132.54 ± 0.95	131.77 ± 1.35	131.87 ± 3.76	0.072
DBP (mean ± SE), mmHg	70.64 ± 0.40	71.46 ± 0.62	68.45 ± 0.69	72.23 ± 1.11	71.87 ± 1.82	0.99
TC (mean ± SE), mmol/L	4.98 ± 0.03	4.76 ± 0.04	4.85 ± 0.04	5.60 ± 0.08	5.87 ± 0.20	< 0.001
HDL (mean ± SE), mmol/L	1.24 ± 0.01	1.24 ± 0.01	1.34 ± 0.02	1.03 ± 0.02	1.24 ± 0.06	< 0.001
LDL (mean ± SE), mmol/L	2.84 ± 0.03	2.81 ± 0.04	2.83 ± 0.04	2.85 ± 0.07	3.34 ± 0.19	0.051
Outcomes						
Poor CVH						
Yes	1236 (34.7)	638 (42.7)	236 (15.3)	383 (47.2)	91 (43.5)	< 0.001
No	2320 (65.3)	859 (57.3)	1308 (84.7)	428 (52.8)	117 (56.5)	
CKD						
Yes	678 (16.7)	249 (16.6)	279 (18.1)	128 (15.8)	20 (9.9)	0.325
No	3373 (83.3)	1248 (83.4)	1265 (81.9)	683 (84.2)	188 (90.1)	
Retinopathy						
Yes	550 (24.2)	348 (23.3)	405 (26.2)	148 (18.2)	105 (50.5)	0.010
No	1726 (75.8)	1149 (76.7)	1139 (73.8)	663 (81.8)	103 (49.5)	
NAFLD						
Yes	517 (22.1)	365 (24.4)	255 (16.5)	206 (25.4)	50 (24.0)	0.041
No	1826 (77.9)	1132 (75.6)	1289 (83.5)	605 (74.6)	158 (76.0)	
Advanced liver fibrosis						
Yes	927 (22.8)	446 (29.8)	235 (15.2)	158 (19.5)	39 (18.6)	< 0.001
No	3133 (77.2)	1051 (70.2)	1309 (84.8)	653 (80.5)	169 (81.4)	
All-cause mortality						
Yes	872 (21.6)	212 (14.1)	431 (27.9)	232 (28.6)	50 (23.9)	< 0.001
No	3171 (78.4)	1285 (85.9)	1113 (72.1)	579 (71.4)	158 (76.1)	
CVD-related mortality						
Yes	230 (5.7)	60 (4.0)	101 (6.6)	70 (8.6)	10 (4.9)	0.014
No	3813 (94.3)	1437 (96.0)	1443 (93.4)	741 (91.4)	198 (95.1)	
Cancer-related mortality						
Yes	131 (3.2)	37 (2.5)	64 (4.2)	30 (3.7)	3 (1.6)	0.128
No	3912 (96.8)	1460 (97.5)	1480 (95.8)	781 (96.3)	205 (98.4)	

Data are numbers (percentages) unless otherwise indicated. All estimates accounted for complex survey designs

### ORs of diabetes complications among different subgroups

Table 2 listed the ORs of poor cardiovascular health (CVH), CKD, retinopathy, NAFLD and advanced liver fibrosis among different T2D subgroups. The poor CVH prevalence of subgroup B was significantly lower than that of the subgroup A (adj. OR: 0.08, 95%CI: 0.05–0.12), while no significant differences between the other three subgroups (all P > 0.05). In the prevalence of CKD, subgroup B had a lower prevalence than subgroup A (adj. OR: 0.69, 95%CI: 0.48–1.00). Compared with subgroup A, subgroup D had a significantly higher prevalence of retinopathy (adj. OR: 2.94, 95%CI: 1.16–7.48). Among the four subgroups, subgroup B had the lowest prevalence of NAFLD and advanced liver fibrosis (adj. OR:0.64, 95% CI: 0.43–0.95, adj. OR: 0.21, 95% CI: 0.15–0.29, respectively).

**Table 2:** ORs of poor CVH, CKD, retinopathy, NAFLD and advanced liver fibrosis among different T2D subgroups

	Subgroup A	Subgroup B	Subgroup C	Subgroup D
Poor CVH (n = 3556)				
Model 1^a^	1.00	0.23 (0.17, 0.31) < 0.001	1.21 (0.85, 1.74) 0.284	1.09 (0.54, 2.20) 0.798
Model 2^b^	1.00	0.11 (0.08, 0.17) < 0.001	1.28 (0.82, 2.00) 0.273	1.49 (0.68, 3.25) 0.317
Model 3^c^	1.00	0.08 (0.05, 0.12) < 0.001	0.74 (0.39, 1.40) 0.347	0.55 (0.23, 1.31) 0.178
CKD (n = 4051)				
Model 1^a^	1.00	0.53 (0.37, 0.77) 0.001	0.86 (0.55, 1.35) 0.515	0.75 (0.33, 1.69) 0.479
Model 2^b^	1.00	0.55 (0.39, 0.79) 0.001	0.86 (0.55, 1.35) 0.514	0.72 (0.31, 1.67) 0.447
Model 3^c^	1.00	0.69 (0.48, 1.00) 0.049	0.61 (0.30, 1.24) 0.167	0.57 (0.25, 1.29) 0.176
Retinopathy (n = 2276)				
Model 1^a^	1.00	1.05 (0.64, 1.74) 0.833	0.70 (0.40, 1.22) 0.203	3.57 (1.56, 8.15) 0.003
Model 2^b^	1.00	1.07 (0.64, 1.78) 0.799	0.67 (0.39, 1.16) 0.153	3.54 (1.57, 8.01) 0.003
Model 3^c^	1.00	1.18 (0.69, 2.01) 0.545	0.66 (0.32, 1.38) 0.263	2.94 (1.16, 7.48) 0.024
NAFLD (n = 2343)				
Model 1^a^	1.00	0.57 (0.40, 0.82) 0.002	1.02 (0.69, 1.53) 0.905	1.17 (0.45, 3.02) 0.750
Model 2^b^	1.00	0.55 (0.38, 0.80) 0.002	1.01 (0.67, 1.53) 0.948	1.24 (0.48, 3.20) 0.658
Model 3^c^	1.00	0.64 (0.43, 0.95) 0.026	0.83 (0.49, 1.42) 0.492	1.33 (0.51, 3.49) 0.559
Advanced liver fibrosis (n = 4060)				
Model 1^a^	1.00	0.19 (0.14, 0.27) < 0.001	0.48 (0.31, 0.74) 0.001	0.69 (0.24, 2.02) 0.500
Model 2^b^	1.00	0.20 (0.14, 0.27) < 0.001	0.47 (0.31, 0.73) 0.001	0.67 (0.23, 1.96) 0.465
Model 3^c^	1.00	0.21 (0.15, 0.29) < 0.001	0.68 (0.31, 1.50) 0.342	0.91 (0.29, 2.88) 0.868

^a^Model 1: adjusted for age, sex and race/ethnicity

^b^Model 2: further adjusted (from Model 1) for education level, family income-poverty ratio, smoking status, ideal physical activity

^c^Model 3: further adjusted (from Model 2) for duration of diabetes, diabetes medication use, self-reported hypertension, hypercholesterolemia, and CVD, hypertriglyceridemia, self-reported hypertension, hypercholesterolemia medication use, systolic blood pressure, diastolic blood pressure, total cholesterol, high density lipoprotein cholesterol, and low density lipoprotein cholesterol

### HRs of all-cause, CVD and cancer-related mortalities of different subgroups

**Fig. 2:**
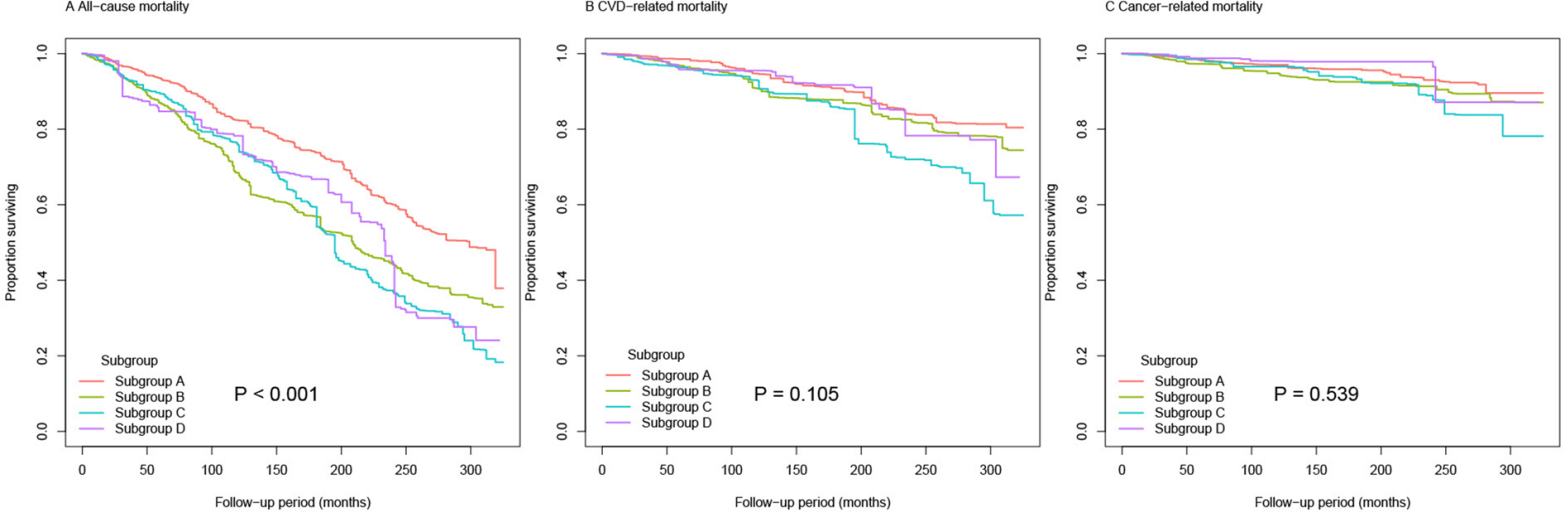
Kaplan–Meier curves for all-cause, CVD and cancer-related mortality categorized by different subgroups of T2D

During 41,447 person-years of follow-up, 1,714 deaths were documented, including 524 CVD-related deaths and 268 cancer-related deaths. Figure 2 showed the Kaplan–Meier curves of the survival rate among the four subgroups, and the cumulative incidence of death due to all-cause was significantly different (log-rank test, P < 0.001). HRs of all-cause, CVD and cancer-related mortality across T2D subgroups were summarized in Table 3. In all-cause mortality, the adjusted HRs and 95% CIs for Subgroup B and Subgroup C were 1.30 (95% CI, 1.02–1.67) and 1.48 (95% CI, 1.06–2.06), respectively, significantly lower than Subgroup A. Subgroup C had the highest, while subgroup A had the lowest all-cause mortality. In CVD and cancer-related mortality, there were no significant difference among the four subgroups (all P > 0.05).

**Table 3:** HRs of all-cause, CVD and cancer-related mortality among different T2D subgroups

	Subgroup A	Subgroup B	Subgroup C	Subgroup D
All-cause mortality (n = 4043)				
Model 1^a^	1.00	1.23 (0.97,1.55) 0.087	1.54 (1.16,2.03) 0.002	1.60 (0.96,2.66) 0.073
Model 2^b^	1.00	1.19 (0.93,1.52) 0.158	1.51 (1.14,1.99) 0.004	1.49 (0.86,2.58) 0.157
Model 3^c^	1.00	1.30 (1.02,1.67) 0.036	1.48 (1.06,2.06) 0.022	1.46 (0.85,2.52) 0.169
CVD (n = 4043)				
Model 1^a^	1.00	0.93 (0.60,1.45) 0.747	1.57 (0.92,2.69) 0.097	1.13 (0.13,10.15) 0.910
Model 2^b^	1.00	0.91 (0.57,1.44) 0.688	1.54 (0.88,2.68) 0.127	1.06 (0.10,10.73) 0.962
Model 3^c^	1.00	1.04 (0.68,1.62) 0.846	1.19 (0.62,2.27) 0.604	0.90 (0.09,9.00) 0.929
Cancer (n = 4043)				
Model 1^a^	1.00	1.03 (0.55,1.96) 0.920	1.19 (0.51,2.78) 0.687	0.69 (0.09,5.15) 0.718
Model 2^b^	1.00	1.02 (0.54,1.92) 0.952	1.14 (0.48,2.67) 0.768	0.66 (0.08,5.19) 0.696
Model 3^c^	1.00	1.11 (0.55,2.24) 0.774	1.43 (0.48,4.25) 0.517	0.76 (0.07,8.02) 0.816

^a^Model 1: adjusted for age, sex and race/ethnicity

^b^Model 2: further adjusted (from Model 1) for education level, family income-poverty ratio, smoking status, ideal physical activity

^c^Model 3: further adjusted (from Model 2) for duration of diabetes, diabetes medication use, self-reported hypertension, hypercholesterolemia, and CVD, hypertriglyceridemia, self-reported hypertension, hypercholesterolemia medication use, systolic blood pressure, diastolic blood pressure, total cholesterol, high density lipoprotein cholesterol, and low density lipoprotein cholesterol

### Subgroup analysis

Additional file 1: Tables S3 and S4 showed stratified analysis of the association between subgroups and complications and mortalities, respectively. In the prevalence of poor CVH, CKD, retinopathy, the results were consistent when analysis were stratified by gender, race and diabetes stages (all P interaction > 0.05). In terms of NAFLD and advanced liver fibrosis, the P interaction with gender, race and diabetes stages were 0.679, 0.031, 0.411 and 0.029, 0.249, 0.791, respectively. The results showed that the prevalence of NAFLD in subgroups B and C were significantly lower than subgroup A in non-white samples. In male samples, the prevalence of advanced liver fibrosis in subgroup D was significantly lower than subgroup A. Regarding mortality risks, except for diabetes stages and all-cause mortality (P interaction = 0.024), no significant interaction was found (all P interaction > 0.05). After excluding the participants who died during the two-year follow-up period, the results of sensitivity analysis were generally consistent with Table 3, as shown in table S5.

## Discussion

Our study proposed a novel yet simple approach to categorize the U.S. T2D population according to the thresholds of FPG, TyG index and BMI. We divided patients into subgroup A (obesity-related diabetes), subgroup B (age-related diabetes), subgroup C (insulin resistant diabetes), and subgroup D (severe insulin deficient diabetes). Patients in subgroup B were older, without severe impaired insulin secretion, severe insulin resistance or obesity. Compared with other subgroups, subgroup B was a low-risk subgroup with the lowest prevalence of poor CVH, NAFLD, and advanced liver fibrosis. NAFLD was closely linked to obesity and insulin resistance [38], as subgroup B had the lowest level of BMI, fasting glucose and insulin resistance, so the risk of NAFLD was low, as well as advanced liver fibrosis. We also found that patients in subgroup B had a higher CVH level than other subgroups. Cardiovascular health (CVH) was related to the risk of CVD [39, 40]. Therefore, although physical function declined with aging, the risk of CVD-related mortality was not very high. Patients in subgroup D had the highest glucose level and severely impaired ability of insulin secretion. The risk of retinopathy in subgroup D was significantly higher than other subgroups, as retinopathy was associated with reduced β-cell function, fasting and postprandial hyperglycemia and hypoinsulinemia instead of insulin resistance [41]. For subgroup C, the all-cause mortality risk was significantly higher than subgroup A, because this subgroup had relatively high levels of age, glucose, BMI and insulin resistance.

From results presented herein, we make the following suggestions: (1) For subgroup A, B and C with a low CVH level, patients should improve diet quality, change dietary behaviors [42] and enhance aerobic exercises [43]; (2) For subgroup D, more attention can be paid to screening for retinopathy; (3) More attention should be paid to all-cause mortality in patients with diabetes, especially for subgroups C; (4) More attention should be paid to screening for NAFLD and advanced liver fibrosis for subgroup A, C and D.

The strengths of this study lie in several aspects. First, we used nine cycles data from the NHANES, which provided a large nationally representative sample of diabetes to analyze. Second, this study conducted a relatively full risk prediction comparison, including the prevalence of CVH, chronic kidney diseases, retinopathy, nonalcoholic fatty liver disease, advanced liver fibrosis, as well as the risks of mortality many years later. Third, the logistic and Cox models were adjusted for several potential confounding factors, including demographic, socioeconomic, lifestyle information, disease history, medication history, etc. However, there are still some limitations. First, as we all know that FPG had a high glycemic variability, and can’t represent islet function stably. It was less stable than HbA1c, but had a stronger correlation with HOMA2-B (β-cell function), as shown by their weighted coefficient: −0.50 for FPG, and −0.36 for HbA1c. This was consistent with the research of Cuiliu Li et al. that FPG showed stronger correlations with indices for β-cell function than HbA1c [44]. In the future, an index similar to TyG index may be developed to represent the function of islet stably. Second, althoughpatients who were diagnosed with diabetes younger than 30 were excluded, type 1 diabetes may also confound our results. In addition, our study has only been validated in the U.S. population, further studies in additional populations are warranted.

## Conclusion

Instead of k-means grouping, we used thresholds of FPG, TyG and BMI to stratify T2D patients into different subgroups. This approach is more practical and easily adopted by clinicians, since it can identify specific subgroup risks, and help clinicians make specific treatment recommendations for people with diabetes. Moreover, fasting glucose, triglycerides and BMI are convenient for detection that can be performed in the primary medical care setting, which has implications for appropriate management, and will go a long way in reducing the complications.

## Supplementary Information

**Additional file 1:** **Table S1.** AHA definitions of cardiovascular health by each metric. **Table S2.** The formula for United States Steatosis Index (USFLI) , fibrosis-4 (FIB-4) and the NAFLD fibrosis score (NFS). **Table S3.** Stratified analysis of the associations (ORs) between different T2D subgroups. **Table S4.** Stratified analysis of the associations (HRs) between different T2D subgroups. **Table S5.** HRs of all-cause, CVD and cancer-related mortality among different T2D subgroups after excluding participants who died within two years of follow-up.

## Data Availability

The NHANES database is available at: https://www.cdc.gov/nchs/nhanes/about_nhanes.htm.

## Abbreviations

BMI: Body mass index
CVD: Cardiovascular disease
CKD: Chronic kidney disease
NAFLD: Nonalcoholic fatty liver disease
NFS: NAFLD fibrosis score
USFLI: United States Steatosis Index
FIB-4: Fibrosis-4
CVH: Cardiovascular health
eGFR: Estimated glomerular filtration rate
FPG: Fasting plasma glucose
HbA1c: Glycated haemoglobin
HRs: Hazard ratios
LS7: The American Heart Association's Life's Simple 7
MARD: Mild age-related diabetes;
MOD: Mild obesity-related diabetes
NHANES: National Health and Nutrition Examination Survey
ORs: Odds ratios
SIDD: Severe insulin-deficient diabetes
SIRD: Severe insulin-resistant diabetes
T2D: Type 2 diabetes
TyG: Triglyceride-glucose index
