# Supplementary material for: An affordable approach to classifying type 2 diabetes based on fasting plasma glucose, TyG index and BMI: a retrospective cohort study of NHANES Data from 1988 to 2014
Source: Diabetol Metab Syndr. 2022 Aug 10;14:113. doi: 10.1186/s13098-022-00883-0 (PMC9364489; doi:10.1186/s13098-022-00883-0)
Supplement: Supplementary file 1 — Additional file 1: Table S1. AHA definitions of cardiovascular health by each metric. Table S2. The formula for United States Steatosis Index (USFLI) , fibrosis-4 (FIB-4) and the NAFLD fibrosis score (NFS). Table S3. Stratified analysis of the associations (ORs) between different T2D subgroups. Table S4. Stratified analysis of the associations (HRs) between different T2D subgroups. Table S5. HRs of all-cause, CVD and cancer-related mortality among different T2D subgroups after excluding participants who died within two years of follow-up. [file 13098_2022_883_MOESM1_ESM.doc]

**An** **affordable approach to classifying type 2 diabetes based on fasting plasma glucose, TyG index and BMI: a retrospective cohort study of NHANES Data from 1988 to 2014**

# Table S1 AHA definitions of cardiovascular health by each metric

| **Health Metric** | **Level** | **Definition** |
| --- | --- | --- |
| **Smoking status ^(a)^** | Ideal | Smoked less than 100 cigarettes |
|  | Intermediate | Smoked at least 100 cigarettes, but currently not smoking at all |
|  | Poor | Smoked at least 100 cigarettes and still smoking |
| **BMI ^(b)^** | Ideal | <25 kg/m^2^ |
|  | Intermediate | 25-29 kg/m^2^ |
|  | Poor | ≥30 kg/m^2^ |
| **Physical activity ^(c)^** | Ideal | ≥150 min/week moderate or ≥75 min/week vigorous  or ≥150 min/week moderate + vigorous |
|  | Intermediate | 1-149 min/week moderate or 1-74 min/week vigorous or 1-149 min/week moderate + vigorous |
|  | Poor | None |
| **Healthy diet score ^(d)^** | Ideal | ≥81 |
|  | Intermediate | 51-80 |
|  | Poor | <=50 |
| **Blood pressure ^(e)^** | Ideal | SBP<120/DBP<80 mmHg |
|  | Intermediate | SBP 120-139 or DBP 80-89 mmHg or treated^(h)^ to SBP<120/DBP<80 mmHg |
|  | Poor | SBP ≥140 or DBP ≥90 mmHg or treated^(h)^ not to SBP<120/DBP<80 mmHg |
| **Total cholesterol ^(f)^** | Ideal | <200 mg/DL |
|  | Intermediate | 200-239 mg/DL or treated^(h)^ to <200 mg/DL |
|  | Poor | ≥240 mg/DL or treated^(h)^ not to <200 mg/DL |
| **FPG ^(g)^** | Ideal | <100 mg/DL |
|  | Intermediate | 100-125 mg/DL or treated^(h)^ to <100 mg/DL |
|  | Poor | ≥126 mg/DL or treated^(h)^ not to <100 mg/DL |

Note:

(a) Cigarette smoking was determined based on self-reports “Smoked at least 100 cigarettes in life” and “Do you now smoke cigarettes”.

(b) BMI (kg/m2) was calculated based on objectively measured by weight and height.

(c) Physical activity was assessed by self-reported frequency and duration of moderate- and vigorous-intensity recreational activities.

(e) Dietary intake was measured from 24-h dietary as recalled by interviewers.

(f) Systolic (SBP) and diastolic (DBP) blood pressures were measured four times consecutively by certified examiners. The average of all available blood pressure measures for each person was calculated.

(g) Total cholesterol (mg/DL) and FPG (fasting plasma glucose) (mg/DL) were assessed using fasting blood samples.

(h) Self-reported history of medications for hypertension, diabetes, and high cholesterol were also obtained to determine whether participants received treatments for above conditions, based on the question “are you now taking prescribed medicines for high blood pressure/cholesterol/blood sugar.”

# Table S2 The formula for United States Steatosis Index (USFLI) , fibrosis-4 (FIB-4) and the NAFLD fibrosis score (NFS)

| **ID** | **Formula** |
| --- | --- |
| (1) | 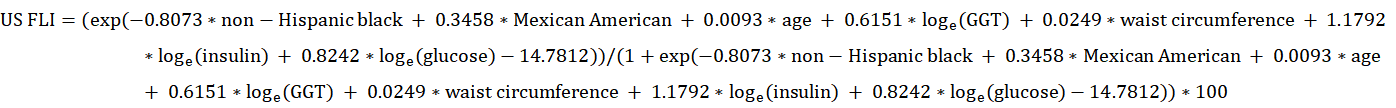 |
| (2) | 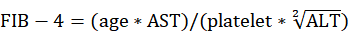 |
| (3) | 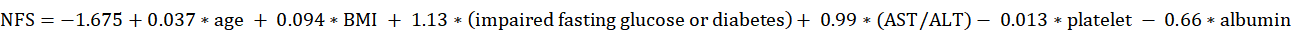 |

Note:

Where “non-Hispanic Black” and “Mexican American” have a value of 1 if the person is of that ethnicity and 0 if the person is not; Where impaired fasting glucose/diabetes has a value of 1 if the subjects have impaired fasting glucose or diabetes and a value of 0 if they do not; age (years), BMI (kg/m2), waist circumference (cm), glucose (mg/dL), insulin (pmol/L), GGT (U/L), ALT (U/L), platelet (×10^9^/L), albumin (g/dL).

# Table S3 Stratified analysis of the associations (ORs) between different T2D subgroups

|  | **Subgroup A** | **Subgroup B** | **Subgroup C** | **Subgroup D** | **P interaction** |
| --- | --- | --- | --- | --- | --- |
| **Poor CVH** | | | | | |
| Gender |  |  |  |  | 0.068 |
| Male (n=1742) | 1.00 | 0.04 (0.02, 0.08) <0.001 | 0.36 (0.15, 0.85) 0.021 | 0.09 (0.02, 0.33) <0.001 |  |
| Female (n=1814) | 1.00 | 0.12 (0.07, 0.20) <0.001 | 1.51 (0.62, 3.67) 0.361 | 2.46 (0.93, 6.51) 0.070 |  |
| Race/ethnicity |  |  |  |  | 0.104 |
| White (n=1236) | 1.00 | 0.08 (0.04, 0.14) <0.001 | 0.77 (0.33, 1.78) 0.532 | 0.88 (0.28, 2.83) 0.831 |  |
| Non-White (n=2320) | 1.00 | 0.06 (0.04, 0.10) <0.001 | 0.61 (0.26, 1.42) 0.247 | 0.26 (0.07, 1.04) 0.057 |  |
| Stages of diabetes |  |  |  |  | 0.959 |
| Newly (n=1236) | 1.00 | 0.08 (0.04, 0.14) <0.001 | 0.56 (0.19, 1.70) 0.303 | 0.37 (0.11, 1.27) 0.112 |  |
| Already (n=2320) | 1.00 | 0.06 (0.03, 0.11) <0.001 | 0.93 (0.43, 2.00) 0.853 | 0.52 (0.18, 1.50) 0.223 |  |
| **CKD** | | | | | |
| Gender |  |  |  |  | 0.312 |
| Male (n=2027) | 1.00 | 1.01 (0.59, 1.72) 0.970 | 0.68 (0.23, 1.98) 0.471 | 0.86 (0.33, 2.25) 0.750 |  |
| Female (n=2024) | 1.00 | 0.50 (0.29, 0.85) 0.011 | 0.46 (0.22, 1.00) 0.049 | 0.47 (0.17, 1.32) 0.149 |  |
| Race/ethnicity |  |  |  |  | 0.953 |
| White (n=1368) | 1.00 | 0.56 (0.33, 0.96) 0.035 | 0.57 (0.24, 1.33) 0.189 | 0.44 (0.16, 1.20) 0.106 |  |
| Non-White (n=2683) | 1.00 | 0.80 (0.54, 1.19) 0.276 | 0.56 (0.20, 1.61) 0.280 | 0.76 (0.24, 2.39) 0.630 |  |
| Stages of diabetes |  |  |  |  | 0.803 |
| Newly (n=1450) | 1.00 | 1.11 (0.60, 2.05) 0.744 | 0.27 (0.10, 0.69) 0.007 | 0.70 (0.11, 4.42) 0.698 |  |
| Already (n=2601) | 1.00 | 0.58 (0.38, 0.88) 0.011 | 0.71 (0.30, 1.68) 0.428 | 0.44 (0.20, 0.98) 0.045 |  |
| **Retinopathy** | | | | | |
| Gender |  |  |  |  | 0.275 |
| Male (n=1058) | 1.00 | 1.05 (0.42, 2.64) 0.907 | 0.60 (0.18, 1.96) 0.392 | 1.23 (0.25, 6.00) 0.798 |  |
| Female (n=1218) | 1.00 | 1.36 (0.61, 3.07) 0.445 | 0.84 (0.27, 2.64) 0.763 | 5.68 (1.99, 16.22) 0.002 |  |
| Race/ethnicity |  |  |  |  | 0.459 |
| White (n=771) | 1.00 | 1.21 (0.52, 2.83) 0.654 | 0.71 (0.29, 1.73) 0.435 | 2.78 (0.81, 9.48) 0.099 |  |
| Non-White (n=1505) | 1.00 | 1.27 (0.68, 2.36) 0.442 | 0.48 (0.15, 1.52) 0.207 | 3.19 (1.04, 9.74) 0.042 |  |
| Stages of diabetes |  |  |  |  | 0.743 |
| Newly (n=832) | 1.00 | 0.82 (0.25, 2.64) 0.733 | 1.19 (0.22, 6.43) 0.834 | 0.74 (0.07, 8.05) 0.803 |  |
| Already (n=1444) | 1.00 | 1.18 (0.69, 2.01) 0.542 | 0.51 (0.17, 1.51) 0.217 | 2.78 (1.08, 7.15) 0.034 |  |
| **NAFLD** | | | | | |
| Gender |  |  |  |  | 0.679 |
| Male (n=1256) | 1.00 | 0.66 (0.43, 1.01) 0.054 | 0.84 (0.42, 1.69) 0.622 | 0.52 (0.19, 1.43) 0.200 |  |
| Female (n=1087) | 1.00 | 0.48 (0.21, 1.09) 0.081 | 0.89 (0.35, 2.22) 0.797 | 2.80 (0.71, 10.95) 0.138 |  |
| Race/ethnicity |  |  |  |  | 0.031 |
| White (n=824) | 1.00 | 0.84 (0.51, 1.38) 0.485 | 1.05 (0.47, 2.36) 0.906 | 2.30 (0.58, 9.13) 0.230 |  |
| Non-White (n=1519) | 1.00 | 0.35 (0.22, 0.56) <0.001 | 0.48 (0.25, 0.92) 0.027 | 0.75 (0.32, 1.76) 0.504 |  |
| Stages of diabetes |  |  |  |  | 0.411 |
| Newly (n=784) | 1.00 | 0.62 (0.33, 1.15) 0.129 | 1.17 (0.42, 3.26) 0.761 | 0.98 (0.30, 3.20) 0.973 |  |
| Already (n=1559) | 1.00 | 0.61 (0.36, 1.03) 0.062 | 0.65 (0.32, 1.32) 0.233 | 1.47 (0.46, 4.70) 0.511 |  |
| **Advanced liver fibrosis** | | | | | |
| Gender |  |  |  |  | 0.029 |
| Male (n=2029) | 1.00 | 0.18 (0.11, 0.30) <0.001 | 0.42 (0.16, 1.10) 0.075 | 0.22 (0.06, 0.81) 0.024 |  |
| Female (n=2031) | 1.00 | 0.24 (0.15, 0.38) <0.001 | 1.21 (0.50, 2.93) 0.675 | 1.97 (0.54, 7.27) 0.304 |  |
| Race/ethnicity |  |  |  |  | 0.249 |
| White (n=1370) | 1.00 | 0.21 (0.12, 0.35) <0.001 | 0.77 (0.28, 2.09) 0.598 | 1.03 (0.15, 6.92) 0.973 |  |
| Non-White (n=2690) | 1.00 | 0.18 (0.12, 0.28) <0.001 | 0.56 (0.24, 1.30) 0.174 | 0.77 (0.33, 1.82) 0.547 |  |
| Stages of diabetes |  |  |  |  | 0.791 |
| Newly (n=1452) | 1.00 | 0.27 (0.16, 0.47) <0.001 | 0.62 (0.22, 1.73) 0.354 | 0.69 (0.13, 3.75) 0.668 |  |
| Already (n=2608) | 1.00 | 0.17 (0.11, 0.27) <0.001 | 0.70 (0.26, 1.87) 0.471 | 0.97 (0.23, 4.07) 0.972 |  |

Note:

All the model adjusted for age, sex, race/ethnicity, BMI, education level, family income-poverty ratio, smoking status, ideal physical activity, duration of diabetes, diabetes medication use, self-reported hypertension, hypercholesterolemia, and CVD, hypertriglyceridemia, self-reported hypertension, hypercholesterolemia medication use, systolic blood pressure, diastolic blood pressure, total cholesterol, high density lipoprotein cholesterol, and low density lipoprotein cholesterol, with exception of stratifying factors.

# Table S4 Stratified analysis of the associations (HRs) between different T2D subgroups

|  | **Subgroup A** | **Subgroup B** | **Subgroup C** | **Subgroup D** | **P interaction** |
| --- | --- | --- | --- | --- | --- |
| **All cause mortality** | | | | | |
| Gender |  |  |  |  | 0.925 |
| Male (n=2017) | 1.00 | 1.22 (0.85,1.75) 0.283 | 1.69 (0.99,2.88) 0.056 | 1.54 (0.66,3.60) 0.321 |  |
| Female (n=2026) | 1.00 | 1.32 (0.90,1.93) 0.154 | 1.28 (0.71,2.33) 0.410 | 1.15 (0.47,2.83) 0.756 |  |
| Race/ethnicity |  |  |  |  | 0.867 |
| White (n=1368) | 1.00 | 1.42 (1.06,1.90) 0.018 | 1.42 (0.91,2.21) 0.120 | 1.38 (0.74,2.55) 0.309 |  |
| Non-White (n=2675) | 1.00 | 1.13 (0.81,1.59) 0.473 | 1.74 (0.91,3.32) 0.094 | 1.53 (0.73,3.22) 0.260 |  |
| Stages of diabetes |  |  |  |  | 0.024 |
| Newly (n=1439) | 1.00 | 1.20 (0.78,1.85) 0.401 | 1.26 (0.70,2.26) 0.448 | 0.46 (0.06,3.34) 0.442 |  |
| Already (n=2604) | 1.00 | 1.39 (1.02,1.88) 0.037 | 1.65 (1.08,2.53) 0.022 | 2.39 (1.44,3.99) 0.001 |  |
| **CVD-related mortality** | | | | | |
| Gender |  |  |  |  | 0.996 |
| Male (n=2017) | 1.00 | 0.73 (0.34,1.60) 0.439 | 1.25 (0.55,2.80) 0.595 | 0.28 (0.00,25.74) 0.580 |  |
| Female (n=2026) | 1.00 | 1.45 (0.58,3.59) 0.428 | 1.08 (0.32,3.59) 0.905 | 2.05 (0.14,30.89) 0.603 |  |
| Race/ethnicity |  |  |  |  | 0.723 |
| White (n=1368) | 1.00 | 1.25 (0.68,2.31) 0.470 | 1.04 (0.49,2.23) 0.912 | 0.89 (0.32,2.49) 0.827 |  |
| Non-White (n=2675) | 1.00 | 0.71 (0.35,1.45) 0.349 | 2.07 (0.77,5.52) 0.148 | 0.98 (0.31,3.10) 0.970 |  |
| Stages of diabetes |  |  |  |  | 0.998 |
| Newly (n=1439) | 1.00 | 1.00 (0.33,3.04) 0.999 | 2.4 (0.88,6.55) 0.086 | 0.68 (0.02,24.90) 0.832 |  |
| Already (n=2604) | 1.00 | 1.06 (0.62,1.81) 0.829 | 0.91 (0.4,2.08) 0.832 | 1.00 (0.33,2.99) 0.999 |  |
| **Cancer-related mortality** | | | | | |
| Gender |  |  |  |  | 0.693 |
| Male (n=2017) | 1.00 | 1.11 (0.46,2.67) 0.808 | 1.22 (0.39,3.87) 0.731 | 0.93 (0.10,8.89) 0.949 |  |
| Female (n=2026) | 1.00 | 0.90 (0.35,2.32) 0.828 | 1.9 (0.41,8.88) 0.415 | 0.51 (0.02,13.16) 0.682 |  |
| Race/ethnicity |  |  |  |  | 0.851 |
| White (n=1368) | 1.00 | 1.35 (0.55,3.28) 0.510 | 1.54 (0.52,4.55) 0.439 | 0.82 (0.00,183.13) 0.943 |  |
| Non-White (n=2675) | 1.00 | 0.56 (0.25,1.28) 0.169 | 1.35 (0.54,3.38) 0.516 | 0.65 (0.12,3.56) 0.616 |  |
| Stages of diabetes |  |  |  |  | 0.641 |
| Newly (n=1439) | 1.00 | 1.89 (0.87,4.13) 0.109 | 2.03 (0.63,6.56) 0.237 | 0.03 (0.00,6.4e+62) 0.963 |  |
| Already (n=2604) | 1.00 | 0.86 (0.35,2.11) 0.744 | 1.31 (0.23,7.37) 0.759 | 1.88 (0.28,12.61) 0.514 |  |

Note:

All the model adjusted for age, sex, race/ethnicity, BMI, education level, family income-poverty ratio, smoking status, ideal physical activity, duration of diabetes, diabetes medication use, self-reported hypertension, hypercholesterolemia, and CVD, hypertriglyceridemia, self-reported hypertension, hypercholesterolemia medication use, systolic blood pressure, diastolic blood pressure, total cholesterol, high density lipoprotein cholesterol, and low density lipoprotein cholesterol, with exception of stratifying factors.

# Table S5 HRs of all-cause, CVD and cancer-related mortality among different T2D subgroups after excluding participants who died within two years of follow-up

|  | **Subgroup A** | **Subgroup B** | **Subgroup C** | **Subgroup D** |
| --- | --- | --- | --- | --- |
| All-cause mortality (n=3689) |  |  |  |  |
| Model 1^a^ | 1.00 | 1.25 (0.95,1.66) 0.110 | 1.53 (1.11,2.12) 0.009 | 1.65 (0.95,2.89) 0.077 |
| Model 2^b^ | 1.00 | 1.21 (0.92,1.61) 0.172 | 1.50 (1.07,2.10) 0.018 | 1.53 (0.85,2.75) 0.155 |
| Model 3^c^ | 1.00 | 1.33 (0.99,1.79) 0.062 | 1.62 (1.07,2.45) 0.023 | 1.68 (0.87,3.24) 0.124 |
| CVD-related mortality (n=3689) |  |  |  |  |
| Model 1^a^ | 1.00 | 0.91 (0.54,1.56) 0.742 | 1.33 (0.71,2.49) 0.371 | 1.05 (0.18,5.99) 0.958 |
| Model 2^b^ | 1.00 | 0.89 (0.52,1.53) 0.680 | 1.29 (0.69,2.43) 0.427 | 0.97 (0.15,6.16) 0.975 |
| Model 3^c^ | 1.00 | 1.01 (0.60,1.71) 0.974 | 1.13 (0.54,2.38) 0.739 | 1.03 (0.19,5.42) 0.974 |
| CVD-related mortality (n=3689) |  |  |  |  |
| Model 1^a^ | 1.00 | 1.12 (0.57,2.19) 0.741 | 1.40 (0.73,2.70) 0.310 | 0.77 (0.03,19.93) 0.874 |
| Model 2^b^ | 1.00 | 1.10 (0.57,2.13) 0.770 | 1.32 (0.67,2.62) 0.421 | 0.72 (0.03,18.22) 0.845 |
| Model 3^c^ | 1.00 | 1.27 (0.65,2.48) 0.476 | 1.93 (0.67,5.54) 0.220 | 1.00 (0.03,31.55) 0.999 |

Note:

a Model 1: adjusted for age, sex and race/ethnicity;

b Model 2: further adjusted (from Model 1) for education level, family income-poverty ratio, smoking status, ideal physical activity;

c Model 3: further adjusted (from Model 2) for duration of diabetes, diabetes medication use, self-reported hypertension, hypercholesterolemia, and CVD, hypertriglyceridemia, self-reported hypertension, hypercholesterolemia medication use, systolic blood pressure, diastolic blood pressure, total cholesterol, high density lipoprotein cholesterol, and low density lipoprotein cholesterol.
